# CO_2_-free Fischer–Tropsch synthesis enabled by halogen modifiers

**DOI:** 10.1093/nsr/nwaf549

**Published:** 2025-11-29

**Authors:** Javier Pérez-Ramírez

**Affiliations:** Institute for Chemical and Bioengineering, Department of Chemistry and Applied Biosciences, ETH Zürich, Switzerland; NCCR Catalysis, Switzerland

Fischer–Tropsch synthesis (FTS) remains the industrial workhorse for converting synthesis gas (CO + H_2_) into hydrocarbons, yet its efficiency is constrained by the substantial CO_2_ emissions characteristic of iron-based catalysts. In a landmark study, Ma, Wen, Liu and co-workers show that introducing halogenated compounds in trace amounts transforms Fe-based FTS, enabling near-zero CO_2_ selectivity while simultaneously increasing olefin productivity [1]. This simple strategy offers a compelling advance toward the carbon-neutral synthesis of fuels and light olefins from syngas.

Iron catalysts dominate commercial FTS processes because of their abundance, tunable product slate and high space–time yields, yet they unavoidably drive the water-gas-shift (WGS) and Boudouard reactions, generating carbon dioxide as a major byproduct and wasting valuable carbon feedstock. Previous mitigation strategies, including hydrophobic coatings or phase-pure carbides [2,3], offered only limited improvement. Ma, Wen, Liu and colleagues demonstrate that cofeeding 20 ppm of bromomethane (CH_3_Br) to a χ-Fe_5_C_2_ catalyst suppresses CO_2_ selectivity from >30% to <1% while increasing the olefin selectivity to ∼85% and increasing the olefin/paraffin ratio by an order of magnitude [1].

In-depth analyses reveal that surface-bound halogens, rather than bulk incorporation, are responsible for this transformation. Spectroscopic and microscopic techniques show that bromine is uniformly distributed on the surface of iron carbide and oxide domains, forming Fe–Br species. Transient kinetic studies and isotopic labeling experiments, complemented by density functional theory simulations, demonstrate that these surface halogens selectively block key elementary steps leading to CO_2_ and paraffin formation: (i) H_2_O dissociation in the WGS pathway, (ii) CO*+O* recombination in the Boudouard reaction and (iii) olefin hydrogenation on carbide surfaces.

Importantly, the catalyst sustained >450 hours of continuous operation with stable CO conversion (∼35%), C_2+_ olefin selectivity of >80% and CO_2_ selectivity of <1.5%, highlighting the remarkable durability and industrial viability. Besides, only trace bromine (<1 ppm) remains in the liquid products, indicating minimal contamination risk. Notably, the approach generalizes to multiple iron carbide phases (ε-Fe_2_C, θ-Fe_3_C, η-Fe_7_C_3_) and even commercial catalysts, underscoring its robustness.

Beyond its catalytic impact, this work [1] establishes a paradigm shift in carbon-efficient syngas conversion, in which halogen-modified catalytic surfaces can steer impressive selectivity changes. The simplicity of ppm-level halogen cofeeding, reminiscent of chlorine promoters in commercial ethylene epoxidation, makes the strategy translatable to large-scale Fischer–Tropsch units.

When coupled with CO_2_-neutral syngas generation (via CO_2_ reforming and green hydrogen), it could enable carbon-neutral or even carbon-negative production of olefins and fuels, bridging the gap between fossil-derived and eventual circular chemical manufacturing. It represents a milestone in the 100-year evolution of the Fischer–Tropsch process, transforming century-old technology into a modern low-carbon platform for sustainable chemical manufacturing.

Further mechanistic studies to elucidate the catalyst structure and dynamic behavior of bromine under operating conditions, alongside long-term stability tests in industrially relevant FTS reactors, will be essential to advance this promising strategy toward deployment. In addition, dedicated investigations into the environmental, safety and materials-compatibility aspects of CH_3_Br (and other halogenated compounds) handling at scale will be critically important.
